# Dose-Dependent Pain and Pain Radiation after Chemical Stimulation of the Thoracolumbar Fascia and Multifidus Muscle: A Single-Blinded, Cross-Over Study Revealing a Higher Impact of Fascia Stimulation

**DOI:** 10.3390/life12030340

**Published:** 2022-02-25

**Authors:** Simon Vogel, Walter Magerl, Rolf-Detlef Treede, Andreas Schilder

**Affiliations:** 1 Mannheim Center for Translational Neuroscience (MCTN), Department of Neurophysiology, Medical Faculty Mannheim, Heidelberg University, Ludolf-Krehl-Straße 13-17, 68167 Mannheim, Germany; vogel.simon@hotmail.de (S.V.); walter.magerl@medma.uni-heidelberg.de (W.M.); rolf-detlef.treede@medma.uni-heidelberg.de (R.-D.T.); 2 Department of Experimental Orthopedics and Trauma Surgery, Medical Faculty Mannheim, Heidelberg University, Theodor-Kutzer-Ufer 1-3, 68167 Mannheim, Germany

**Keywords:** chemical stimulation, hypertonic saline, thoracolumbar fascia, multifidus muscle, pain intensity, pain distribution, peripheral sensitization, low back pain

## Abstract

Acute low back pain can be experimentally induced by injections of hypertonic saline into deep tissues of the back, such as fascia and muscle. The current study investigated the dose-dependency of peak-pain and spatial extent of concomitant radiating pain following 50, 200 and 800 μL bolus injections of hypertonic saline (5.8%) into the thoracolumbar fascia and multifidus muscle, since data on dose-dependency is lacking in humans. Sixteen healthy subjects rated (11 female, 5 male; 23.3 ± 3.1 years, mean ± SD) intensity and spatial extent of pain. Injections into the fascia resulted in significantly higher peak-pain (+86%, *p* < 0.001), longer pain durations (*p* < 0.05), and larger pain areas (+65%, *p* < 0.02) and were less variable than intramuscular injections. Peak-pain ratings and pain areas were 2–3-fold higher/larger for 200 μL vs. 50 μL. In contrast, peak pain increased only marginally at 800 μL by additional 20%, while pain areas did not increase further at all in both, fascia and muscle. Thus, higher injection volumes did also not compensate the lower sensitivity of muscle. Peak-pain ratings and pain areas correlated between fascia and muscle (*r* = 0.530, *p* < 0.001 and *r* = 0.337, *p* < 0.02, respectively). Peak-pain ratings and pain areas correlated overall (*r* = 0.490, *p* < 0.0001), but a weak correlation remained when the impact of between-tissue differences and different injection volumes were singled out (partial *r* = 0.261, *p* < 0.01). This study shows dose-dependent pain responses of deep tissues where an injection volume of 200 μL of hypertonic saline is deemed an adequate stimulus for tissue differentiation. We suggest that pain radiation is not simply an effect of increased peripheral input but may afford an individual disposition for the pain radiation response. Substantially higher pain-sensitivity and wider pain areas support fascia as an important contributor to non-specific low back pain.

## 1. Introduction

Although structures of the spine, such as vertebrae or intervertebral discs, are recognized as causes of low back pain (LBP) [1], the role of fascia tissue is gaining increasing scientific attention by identifying or supporting this particular tissue as a potential source of pain [2,3,4,5,6,7,8,9,10,11]. Lumbar dorsal horn neurons receive input from nociceptive free nerve endings [12] located in the thoracolumbar fascia [13,14,15,16]. Furthermore, fascia tissue has been identified to play an important role in the sensitivity to pain stimuli, that fascia is more sensitive to both chemical and electrical stimulation than the underlying muscle leading to higher pain intensities, pain duration, and larger pain distribution [4,5]. The stimulated fascia nerve endings lead to a distinctly higher affective and sharper mechanical pain character compared to other deep tissues [4,6]. They are able to induce longer-lasting pain amplification [5] and we recently revealed a somatosensory crosstalk between deep fascia tissue and superficial cutaneous tissue [7].

Injections of hypertonic saline are a well-validated model of deep tissue pain and are frequently used to excite nociceptors in deep tissues resulting in an activation of the nociceptive system by depolarizing small diameter nociceptive afferent neurons [17], while it blocks the generation of action potentials in large diameter fibers [18]. It has been shown that the thoracolumbar fascia has a three-times higher innervation density than the muscle [9] and that a hypertonic saline injection into the fascia evokes up to 2.5-times higher pain intensities compared to muscle stimulations [4]. Nonetheless, it is unknown if higher evoked pain also results in larger spatial extent of pain when comparing the timepoint of peak pain intensity. This will give insight in the role of spatial summation in mediating ongoing pain in low back pain patients. Since there is no comparative study distinguishing the outcome of pain parameters after different stimulation intensities within the same tissue, this study aims at investigating the chemical sensitivity of the thoracolumbar fascia and of the underlying multifidus muscle to different volumes of hypertonic saline by analyzing pain intensity and spatial extent. Furthermore, we aim at determining the volume of saline recruiting an adequate proportion of nociceptive free nerve endings that allows the differentiation between fasciae and muscles and to determine equipotent stimulation conditions.

We hypothesize that an injection of hypertonic saline into the thoracolumbar fascia and the multifidus muscle will reveal a dose dependent pain perception with the highest pain intensity and the largest pain radiation after fascia stimulation. Furthermore, we expect a positive correlation between the area of pain radiation after chemical stimulation and the elicited pain intensity and the volumes that were injected into these tissues.

## 2. Material and Methods

### 2.1. Participants

Sixteen young healthy volunteers (11 female, five male; 23.3 ± 3.1 years, mean ± SD) with no history of back pain participated in this study. All volunteers signed a written consent form and had sufficient command of German language. The criteria for exclusion were acute or persistent pain, recent surgeries orany type of medication assessed by a structural self-disclosure questionnaire. None of the participants withdrew from the study prematurely. Local ethics committee approval had been obtained according to the current version of the Declaration of Helsinki (Medical Faculty Mannheim, Heidelberg University ethics committee II, 2020-533N). This study was part of another study on skin tenderness induced by electrical or hypertonic saline stimulation of the low back and fasciae regions [7], where we reported only raw study data in brief for explanation. Nonetheless, here, we logarithmic transformed all raw data to achieve secondary normal distribution. For the ease of comprehension, we retransformed the respective log means [19] and put it in relation to the AUC, pain duration and distribution, thus, here we report fully detailed stimulus-response functions of various parameters of deep tissue pain rather than cutaneous sensitivity.

### 2.2. Saline Administration

Bolus injections of hypertonic (5.8%) saline were made into the thoracolumbar fascia (deep fascia [20]) or the underlying multifidus muscle at lumbar level (L3/L4) about 2 cm lateral to the spinous processes using different injection volumes (50 μL, 200 μL and 800 μL). The position of the injection needle for each bolus injection of hypertonic saline was guided by ultrasound (M-Turbo^®^ ultrasound system; Sonosite, Munich, Germany) with a linear transducer (HFL50×/15 MHz Linear Array, Sonosite Transducer). In contrast to fascia injections, saline injections into the muscle were performed vertically about one centimeter beyond the fascia after pulling the skin sideways in order to prevent capillary effects after needle withdrawing probably leading to fluid reflow. The solution was administered using a 1-mL syringe (Becton Dickinson, Madrid, Spain) and a 27 G cannula.

### 2.3. Experimental Protocol

All experiments were conducted in a quiet ambient temperature and humidity-controlled human research laboratory environment between August 25th and December 1st 2020 with a beforehand recruitment period of approximately 2 months.

Subjects were advised to lie face-down on a bench and to abstain from active back muscle contraction. Saline administration was performed as described in previous section.

**Figure 1:**
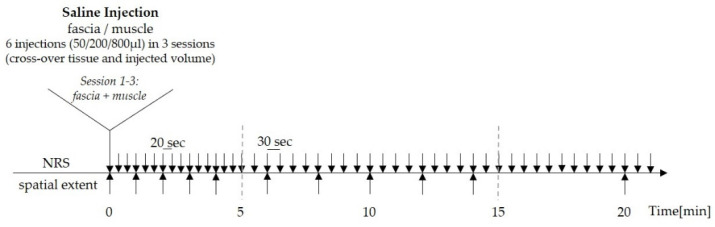
Experimental design of the study. Arrows indicate inquiry of pain intensity rating (NRS) and drawing of pain radiation area (spatial extent) following chemical induced pain. The experimental design was a fully right-left balanced cross-over design comprising the tissue type stimulated by hypertonic saline and the volume of saline injected.

In order to delineate adequate conditions for stimulation intensity to be able to compare different tissues and to timely match peak-pain intensity and spatial extent of evoked pain, the protocol was designed as follows: The volunteers were asked to rate the magnitude of perceived pain at 20 s intervals for the first 5 min, and thereafter at 30 s intervals for the following 20 min (total time of pain assessment was 25 min) on a numerical rating scale (NRS) with the endpoints 0 (= no pain) and 100 (= most intense pain imaginable). Pain was defined as “stinging”, “burning” or “pricking” at any intensity on NRS above 0. While they rated the experimentally induced pain, the subjects also marked the distribution of pain by drawings on a standard human body scheme every 60 s within the first 4 min, then every 120 s for the following 10 min, and then again at 20 min and 25 min after injection until the end of pain perception (Figure 1).

### 2.4. Pain Radiation

All volunteers were asked to localize their acute pain areas on a standardized two-dimensional body image paper form while they perceived the experimentally induced low back pain. In order to compare the spatial extent at the timepoint of maximum pain intensity, i.e., peak-pain rating, the standard human body scheme was presented during the entire 25 min post-injection period but exchanged several times during that time period, as described in the previous section.

### 2.5. Statistics

The necessary number of subjects successfully completing the study, i.e., treated per protocol, was calculated using the open source power analysis software G*Power, release 3.1.9.7 for Windows [21]. For two-tailed comparison and *p* = 0.025 (corrected for two stepwise comparisons of the three different injection volumes), a power of 0.80 and the assumption of a medium-to-large effect size (Cohen’s *d* = 0.65), an estimate that was derived from earlier studies [4,5,6,7], were included in the calculation.

Statistical analysis was performed using SigmaPlot software; version 12.4 (Systat Software, Inc., Inpixon GmbH, 40212 Düsseldorf, Germany). Significant differences (at *p*-values < 0.05) were determined by repeated measures analysis of variance (RM-ANOVA) followed by the Holm-Sidak post hoc test, which controls the family-wise error rate (FWER) using stepwise rejection adjustment combined with Sidak’s correction. It is a modified Bonferroni test (Holm-Bonferroni) with a higher power and a lower increase of type II error risk than the classical Bonferroni method. It assumes independent pairwise comparison and therefore provides the advantage of not necessitating overall homogeneity of variances [22]. Normal distribution was confirmed for all but one outcome parameter (pain radiation following 50 μL of muscle injection). To avoid undue failure to reject the null hypothesis (type II error) the difference of pain radiation between fascia and muscle was retested using the non-parametric Wilcoxon Signed Rank Test.

For the analyses of correlations, the Pearson Product Moment Correlation was used. For correlations of pain parameters between fascia and muscle, the impact of different injection volumes was singled out using partial correlations. All values given in this study are depicted as mean ± SD in the results, and as mean ± SEM in the figures.

Before calculations, the data of pain intensity, area under the pain rating curve (AUC) and spatial extent were transformed into decadic logarithms to achieve secondary normal distribution [19,23]. For easier comprehension, the retransformed log mean, which is equivalent to the geometric mean is reported in the results together with the log mean ± SD of log data.

Regarding the pain drawings of each subject, areas were digitized (600 dpi) and transformed into a color-coded image using MATLAB (The MathWorks, Inc,, Natick, MA, USA). In the group analysis, body areas with high or low occurrence of pain were illustrated in dark red or light yellow, respectively. Body areas without pain appear white in the graphic representations.

## 3. Results

### 3.1. Pain Intensity and Duration after Hypertonic Saline Injection

**Figure 2:**
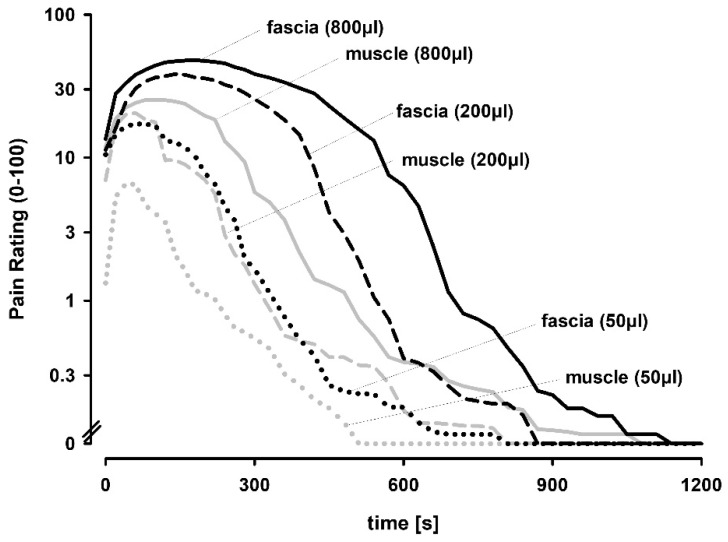
Time courses of pain ratings on a 0–100 numerical rating scale (NRS) for different volumes of hypertonic (5.8%) saline into the fascia and muscle. Injection of hypertonic saline into the fascia or muscle revealed dose-dependent progressive increases of pain (n = 16).

**Figure 3:**
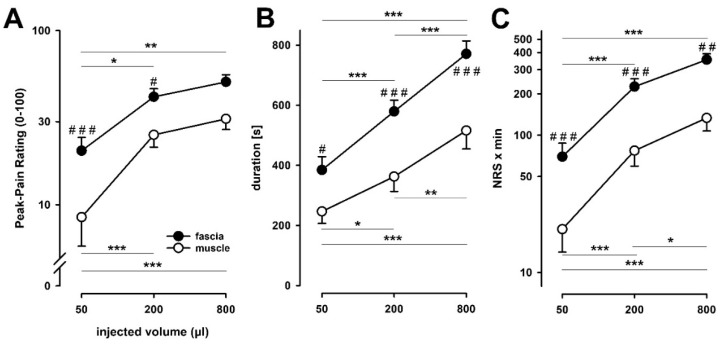
Pain ratings and pain durations after hypertonic saline stimulations of the fascia and muscle. Peak-pain ratings (**A**), pain duration (**B**) and area under the pain rating curve (AUC; (**C**)) after hypertonic saline injections into the fascia (filled circles) and muscle (open circles) are shown. An injection of hypertonic saline into the fascia induced the largest perceived peak-pain compared to a hypertonic saline injection into the muscle. The pain duration after muscle injection of hypertonic saline was reduced compared to fascia injections. (* *p* < 0.05, ** *p* < 0.01, *** *p* < 0.001 within tissues; # *p* < 0.05, ## *p* < 0.01, ### *p* < 0.001 between tissues after hypertonic saline injection; n = 16).

Different volumes of hypertonic saline evoked volume dependent increases of pain responses (Figure 2). Pain intensity ratings increased slowly and reached maximal pain ratings at approximately 1, 2 and 3 min, respectively, for 50, 200 and 800 μL of injected volume. Peak pain after fascia injection occurred later and pain lasted longer than after muscle injections. Likewise, fascia injections yielded stronger peak pain ratings (1.5–2.5-fold higher than muscle), as well as longer pain durations and half-lives (1.5–2-fold longer than muscle). Only 1/96 injections (50 μL) was not painful. Notably, pain responses plateaued at the highest injection volume of 800 μL. Detailed analysis of these aforementioned pain rating parameters revealed quantitative differences between fascia and muscle injections, and between different injection volumes (Figure 3A–C).

By and large, stimulus-response relationships appeared to be shifted in parallel to higher peak pains for fascia injections. This assumption was supported by ANOVA of the peak-pain rating (PPR), which revealed a significant main effect of “tissue” (F = 12.1, *p* < 0.01) and “volume” (F = 17.6, *p* < 0.001) but no interaction between both main effects (F = 1.2, *p* = 0.32; two-way RM ANOVA, Figure 3A). Peak pain in the fascia was consistently higher at all volumes of injection, namely 20.4 vs. 8.5/100 NRS (log NRS: 1.310 ± 0.305 vs. 0.929 ± 0.667, *p* < 0.05) to 50 μL of volume, 41.6 vs. 25.2/100 NRS (log NRS: 1.620 ± 0.186 vs. 1.401 ± 0.281, *p* < 0.01) to 200 μL of volume injections and 50.7 vs. 31.1/100 NRS (log NRS: 1.705 ± 0.166 vs. 1.493 ± 0.245, *p* = 0.001) to 800 μL of volume. Across all injection volumes there was a grand average higher pain sensitivity of the fascia of +86% (difference of log NRS: 0.271 ± 0.424, *p* < 0.0001).

An injection volume of 200 μL was significantly more painful than 50 μL in the muscle (2.97-fold, *p* < 0.001) and in the fascia (2.04-fold, *p* = 0.01). In contrast, the largest volume of 800 μL increased the peak pain only marginally further in the muscle and in the fascia (+22% and +24%, respectively vs. 200 μL), which did, however, not reach significance (*p* = 0.39 and *p* = 0.43, respectively). Across all stimulations, i.e., fascia, muscle and different injection volumes, the peak pain intensity showed a significant correlation with the injected volume (*r* = 0.484, *p* < 0.0001). This was similar in muscle and fascia (*r* = 0.476 and r = 0.581, both *p* < 0.001). Likewise, peak pain intensity in muscle and fascia were strongly correlated (*r* = 0.530, *p* < 0.001). This correlation remained at somewhat decreased strength when the impact of injection volume and of tissue was singled out (partial *r* = 0.485, *p* < 0.001).

Likewise, pain duration depended significantly on both main effects (ANOVA: F = 11.7, *p* < 0.01 for “tissue”) and F = 12.4, *p* < 0.001 for “injection volume”). Again, there was no significant interaction between both (F = 0.7, *p* = 0.50; two-way RM ANOVA, Figure 3B). Larger injection volumes resulted in progressively longer pain durations (*p* < 0.001 for fascia and *p* < 0.05 for muscle, respectively for 200 vs. 50 μL) and after 800 μL (*p* < 0.001 for fascia, *p* < 0.01 for muscle, respectively for 800 vs. 200 μL). Fascia injections yielded significantly longer pain durations compared to the muscle for the corresponding injection volumes (*p* < 0.05 for 50 μL, *p* < 0.001 each for 200 μL and 800 μL). Pain durations also correlated with injected volumes (*r* = 0.516, *p* < 0.0001).

ANOVA on the pain AUC as a integrated global measure of pain (Figure 3C) also revealed a significant main effect of “tissue” (F = 21.5, *p* < 0.001) and “volume” (F = 41.6, *p* < 0.001) but showed no statistically significant interaction between both (F = 0.3, *p* = 0.78; two-way RM ANOVA). The Pain AUC after injections of 200 μL into the fascia and of 800 μL of saline into both the fascia and the muscle was significantly higher than the AUC for 50 μL injections (*p* < 0.001). Additionally, AUC of fascia injections of 50 μL, 200 μL and 800 μL were significantly larger than muscle stimulation (*p* < 0.001, *p* < 0.001, *p* < 0.01, respectively).

### 3.2. Spatial Extent of Pain after Hypertonic Saline Injection

**Figure 4:**
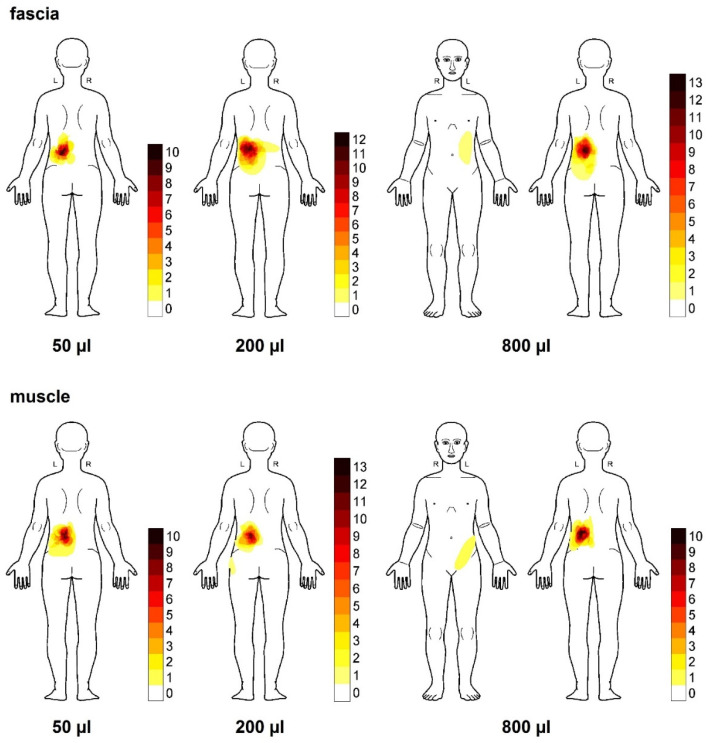
Body chart of individual locations of pain. Super-imposed pain distribution areas after injection of different volumes of hypertonic (5.8%) saline (50/200/800 μL) into the fascia (upper row) and the muscle (bottom row). The body chart represents the timepoint at the individual highest pain evoked by the stimulus (peak-pain rating). The white areas mark body parts without pain in any subjects; the dark red areas and the light-yellow areas mark body parts with high and low occurrence of overlapping pain radiation, respectively reported by all subjects (n = 16).

Hypertonic saline stimulations evoked pain at the point of injection and in adjacent areas. The superimposed spatial distribution of perceived pain at the timepoint of the highest pain intensity individually is shown in Figure 4. The induced low back pain was always located unilaterally to the side of injection with maximal spread onto the midline (in 94/96 injections). In only one subject, who did also not experience pain after 50 μL injection no pain area could be assessed. In only one additional case after injection of 200 μL into the fascia, a subject reported an extension across the midline into the contralateral segment. In addition, pain evoked by larger injection volumes showed a more widespread pain radiation following injection into the fascia (200 and 800 vs. 50 μL). Pain after 50 μL of chemical stimulation was very focal around the injection site, whereas after 200 μL injections, pain radiated into the buttock area in individual subjects, and in addition sometimes into contralateral areas after fascia stimulation.

**Figure 5:**
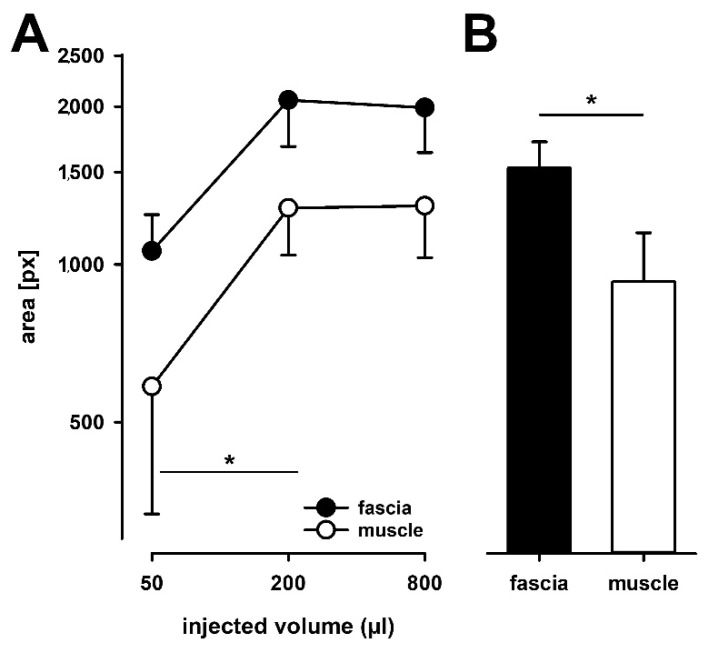
Analysis of the super-imposed pain radiation areas. The analysis of the areas represents the time point at the individual highest pain evoked by the stimulus (peak-pain rating). Pain areas are depicted after different volumes of hypertonic saline injections into the fascia and muscle (**A**) and overall difference between injections into the fascia and the muscle are compared (**B**). The magnitude of pain radiation was calculated in pixels of image resolution. Hypertonic saline injections into the fascia of all subjects resulted in a higher pain radiation pattern compared to muscle stimulations. (* *p* < 0.05, n = 16).

Only in single cases an 800 μL injection into both fascia and muscle tissue elicited pain radiating into ventral areas (one each, but in different subjects). The average pain area after 50 μL was 585 pixels (log10 mean: 2.767 ± 0.973) after muscle injection and almost twice as large after injection into the fascia (1060 pixels; log10 mean: 3.025 ± 0.279; Figure 5A). However, the difference failed to be significant due to the large variability of responses following muscle injection (*p* = 0.097). At higher volumes average pain areas following muscle injections were 1281 pixels (log10 mean: 3.108 ± 0.360) after 200 μL and 1294 pixels (log10 mean: 3.11 ± 0.395) after 800 μL. Following 200 μL and 800 μL injections into the fascia the pain areas were 2058 pixels (log10 mean: 3.313 ± 0.354) and 1990 pixels (log10 mean: 3.11 ± 0.395), respectively. Thus, 800 μL did not yield larger pain radiation areas than 200 μL after either muscle or fascia injection. However, for both injection volumes pain radiation areas were significantly larger after fascia injections (both *p* < 0.05, Figure 5B).

Due to much larger variability of the mapped pain areas compared to NRS pain rating, ANOVA of the pain distributions revealed only a significant trend effect for “tissue” (F = 4.2, *p* = 0.058), but a significant main effect of “volume of injection” (F = 5.3, *p* < 0.01), while there was no interaction at all between both (F = 0.1, *p* = 0.926; two way RM-ANOVA, Figure 5A). Since normality of distribution was also violated, we retested the tissue difference by the non-parametric Wilcoxon Signed Rank Test, which revealed a significant overall difference between fascia and muscle (*p* < 0.05, Figure 5B).

**Figure 6:**
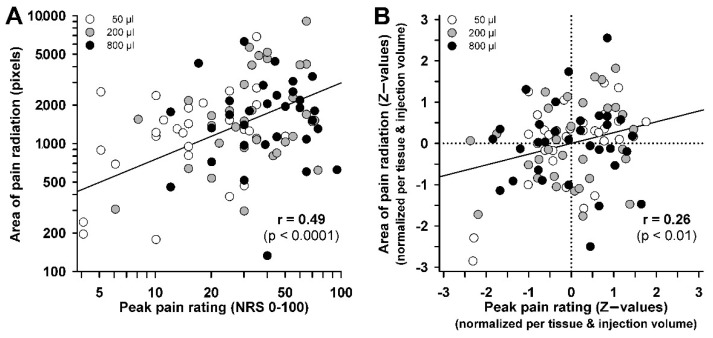
Correlation analysis between spatial extent of pain and peak pain ratings. Correlation analysis of peak pain rating vs. pain radiation areas for raw data (**A**) and for residuals after singling out the impact of different injection volumes and between-tissue difference (**B**). Standard-normalized data that are individually normalized for muscle and fascia, and every injection volume (*Z*-values) depict the true partial correlation devoid of binding by other sources of variance (total n = 96).

Analyses of pain distribution patterns revealed only a weak correlation with injection volume (*r* = 0.208, *p* < 0.05). Pain radiation areas correlated also significantly between fascia and muscle injections (*r* = 0.337, *p* < 0.02). This correlation was even marginally increased when the impact of injection volume was single out (partial *r* = 0.370, *p* < 0.001). Areas of pain radiation correlated strongly with peak pain ratings when calculated across all stimulus conditions (*r* = 0.490, *p* < 0.0001, Figure 6A). However, when the impact of injection volume and tissue was singled out only a weak correlation remained (partial *r* = 0.261, *p* < 0.01, Figure 6B). Since both parameters exhibited <7% of common variance the magnitude of pain rating was only loosely related to the spatial distribution of the pain.

## 4. Discussion

Almost all deep tissues are supplied with considerable densities of nociceptive innervation [24]. Accordingly, soft tissues have been related to the development of low back pain (LBP) [4,5]. It has been shown that the human thoracolumbar fascia is more sensitive to chemical stimulations by 400 μL hypertonic saline than the underlying erector spinae muscle according to peak pain, pain duration and, pain radiation [4]. In the present study, we demonstrate that hypertonic saline injections elicited a graded dose-dependent pain lasting for several minutes with the thoracolumbar fascia being generally more prone to respond to chemical stimulation than the underlying multifidus muscle leading to higher pain scores, longer pain durations and larger pain distribution patterns.

Injections of hypertonic saline into deep tissues have been used for decades to induce deep pain [25] by exciting all group IV afferents [26]. Moreover, microneurography of muscle nerves demonstrated the presence of group III (A-delta) and IV (C-fiber) nociceptors in human muscle [27,28]. Furthermore, it is known that the fascia tissue is densely innervated by nociceptive free nerve endings [13,14,29] and that these mentioned fibers are suggested to be important for detection of chemical stimulation in the lower back [4]. Notably, nociceptive innervation of the fascia is up to three times more densely innervated than muscle [9]. The data that we present in this study demonstrates a dose-dependent pain response in both the thoracolumbar fascia and the underlying multifidus muscle. However, the largest volume (800 μL) elicited only were modest increases compared to 200 μL. This suggests that pain and subsequent pain-related responses plateaued already at the smaller volume. It also supports the interpretation that injection volume is not a dominant component of the pain responses to injection, which confirms previous results using saline injection [4].

It has previously been shown that the use of 400 μL hypertonic saline elicited a pain response that differs between these tissues in pain AUC and peak pain, duration and distribution of pain all being more pronounced following injection into the fascia [4]. The lower pain intensity after muscle stimulation in this study and in recent studies is suggested to be related to the lower density of nociceptive endings in muscle or a less pronounced central representation [12]. A higher sensitivity to painful stimulation of fascia as compared to muscle has also been reported for the crural fascia and tibialis anterior muscle [10]. In detail, the thoracolumbar fascia of mice is shown to be approximately 1.5–3 times more densely innervated by CGRP- and SP-positive nerve endings than the erector spinae muscles or the latissimus dorsi muscle [9], but it is unknown if this factor of a higher innervation density between tissues is directly correlated with the factor of higher pain intensity, even though the pain intensity after fascia stimulations in this study was up to 2.4-times higher than after muscle stimulations. Nonetheless, the differences in perceived pain between the fascia and muscle tissue of the back over all stimulation intensities used in this study may indicate that the thoracolumbar fascia shows a higher primary afferent barrage. This supports its recently summarized potential nociceptive role [8] and suggests the fascia as the more dominant tissue in the development and/or persistency of LBP than the muscle [4]. The dose-dependency of the tissues investigated here, and accordingly, the positive correlation between the elicited pain intensity and the volume injected both verify a high central efficacy of both fascia and muscle input. Nonetheless, even in mild stimulation intensities, the fascia appears to be the most sensitive tissue to experimental stimuli in this study, which supports previous findings [4,5,6].

It has been shown that high dose (400 μL) chemical stimulations of the human thoracolumbar fascia led to significantly longer pain durations compared to stimulations of the underlying multifidus muscle [4]. In this study, we can confirm this significant difference in peak pain, pain duration and pain radiation. The difference was obvious and exhibited a similar magnitude at all injection volumes. However, all pain parameters, with the exception of pain duration plateaued at the two highest volumes, which also suggested that the reduced capacity of muscle injections to elicit pain cannot be overcome by stronger stimuli (i.e., larger injection volumes).

Since our subjects did not move during pain perception, the shorter pain duration after muscle injection is thus not due to active contraction or stretching of the paraspinal muscles shown to suppress pain to intramuscular hypertonic saline injection [30]. In a previous experimental design, control injections of identical volumes of isotonic saline induced only short-lived pain sensations indicating that change in tissue pressure induced by the bolus injection plays a negligible role in the duration of pain [4]. Moreover, pain to control injections did not differ between fascia and muscle. Therefore, variations in tissue compliance are unlikely to explain the differences between fascia tissue and neighboring muscle tissue.

In this study, pain radiation was mostly confined to ipsilateral segments regardless of tissue type or volumes used for chemical stimulation. Similarly, previous results did not show pain radiation to the contralateral side after saline injection into the thoracolumbar fascia or erector spinae muscles [4]. Furthermore, animal studies reported that the receptive fields of spinal dorsal horn neurons of intact rats were strictly located ipsilateral when investigating the thoracolumbar fascia and multifidus muscle [12]. Similar findings were observed in dorsal horn neurons receiving input from other deep tissues of the low back [31]. The painful area after fascia injection was exceeded those after intramuscular injection when using both lower and higher amounts of saline. Thus, differences in pain radiation between fascia and muscle may require only a minimum amount of spatial summation to become apparent. Since there was only a limited correlation between size of pain and volume injected in this experimental design, spatial summation may play only a modest role in mediating ongoing pain in low back pain patients. Furthermore, a positive correlation between the size of pain areas at the timepoint of peak pain and peak pain itself has been shown in this study. This was similarly described after electrical muscle stimulation [32]. These suggest an apparent correlation between size of pain and the areas of pain radiation. However, this may have been caused by the 16-fold variation of injected volume and by differences in sensitivity between muscle and fascia. This is further emphasized when the illusionary inflation of correlation by variable injection volumes or between-tissue differences was removed from the correlation equation. The correlation was then strongly diminished and the common variance was thus very low (<7%), which suggests that pain radiation is not simply an effect of increased peripheral input, but may afford an individual disposition for the pain radiation response. A similar weak coupling has been shown previously for long-term potentiation pain following high frequency electrical stimulation, a measure of nociceptive synaptic plasticity [5]. Future studies may thus concentrate on this factor of individual response disposition for pain plasticity and for pain radiation to understand the high interindividual variability of pain responses following nociceptive events in deep tissue.

## 5. Conclusions

This study has shown that ultrasound-guided injections of several volumes of hypertonic saline into the thoracolumbar fascia and the underlying multifidus muscle induce a dose-dependent pain with evoked distribution patterns similar to acute low back pain.

Compared to muscle stimulations, higher peak-pain levels and concomitant larger pain areas were observed after fascia stimulation using both high and low stimulus intensities, which might be explained by its higher nociceptive innervation and/or the higher afferent barrage. Regarding spatial extent of pain, we report here that the injection volume is not a dominant component of the pain responses to injection. Moreover, we suggest that pain radiation is not simply an effect of increased peripheral input but may afford an individual disposition for the pain radiation response. Regarding the stimulus intensity, evoked pain parameters, such as intensity and distribution pattern, vary between study cohorts [4]. Since differences in pain radiation between fascia and muscle may require a minimum amount of spatial summation to become apparent, we suggest that approximately 200 μL of hypertonic saline stimulates an adequate proportion of nociceptive free nerve endings that allows the differentiation between fasciae and muscles regarding several pain parameters.

Consistent with previous experiments, the fascia is systematically the most pain sensitive deep tissue in the lower back and its innervation may hence play a major role in acute localized low back pain. Since ultrasound guided injections into fascia or muscle are useful for testing their sensitivity, this assumption may be tested by comparing chemically induced pain intensity levels and elicited pain distribution patterns for fascia and muscle input in healthy subjects with those of clinical low back pain patients. Furthermore, it has been shown that the innervation density within several fasciae can differ [33] as well as their central efficacy after hypertonic saline stimulation [7]. Thus, further studies are needed to investigate differences in pain behavior within the fascia network.

## Data Availability

Data can be made available by the author upon request.
